# Young and Older Adults' Gender Stereotype in Multitasking

**DOI:** 10.3389/fpsyg.2015.01922

**Published:** 2015-12-22

**Authors:** Tilo Strobach, Alesia Woszidlo

**Affiliations:** ^1^Medical School Hamburg University of Applied Science and Medical UniversityHamburg, Germany; ^2^Department of Communication Studies, The University of KansasLawrence, KS, USA

**Keywords:** stereotyping, gender stereotyping, multitasking, multitasking performance, cognitive aging

## Abstract

In the present study, we investigated discrepancies between two components of stereotyping by means of the popular notion that women are better at multitasking behaviors: the cognitive structure in individuals (personal belief) and the perceived consensus regarding certain beliefs (perceived belief of groups). With focus on this notion, we examined whether there was empirical evidence for the stereotype's existence and whether and how it was shared among different age groups. Data were collected from 241 young (*n* = 129) and older (*n* = 112) German individuals. The reported perceptions of gender effects at multitasking were substantial and thus demonstrated the existence of its stereotype. Importantly, in young and older adults, this stereotype existed in the perception of attributed characteristics by members of a collective (perceived belief of groups). When contrasting this perceived belief of groups and the personal belief, older adults showed a similar level of conformation of the gender stereotype while young adults were able to differentiate between these perspectives. Thus, young adults showed a discrepancy between the stereotype's components cognitive structure in individuals and perceived consensus regarding certain beliefs.

## Introduction

Stereotypes are cognitive schemata that allow people to make generalizations about individuals based on their specific characteristics, traits, or attributes (Lippmann, 1922; Leyens et al., 1994). The function of stereotyping is to overcome a lack of knowledge and a complex reality, as well as it enables a simplification of social information. The research literature defines stereotyping as (1) an abstract cognitive structure combining facts and information about groups of individuals or (2) the perception of characteristics attributed to target groups by (most of) all members of a collective and thus rather the perception of such an abstract cognitive structure (e.g., Devine, 1989; Nowak et al., 1990; Stangor and Schaller, 2000; Cohen and Garcia, 2005). In other words, stereotypes can have two components: (1) a cognitive structure in individuals (i.e., personal belief) or (2) a perceived consensus regarding certain beliefs (perceived belief of groups). The aim of the present study is to investigate potential discrepancies between both components, conceptualized as potentially generating different outcomes about a particular matter when deliberately taking different perspectives, e.g., personal belief vs. perceived belief of groups. Further, we examine whether these components differ across age groups and ultimately whether gender is a factor that influences multitasking and multitasking performance.

### Stereotyping and aging

A stereotyping phenomenon may be created due to a direct associative pathway through which beliefs can be triggered and expressed (Bodenhausen and Macrae, 1998; Bargh, 2006; Cunningham and Macrae, 2011). As a result, public contexts early in life may set the stage for the automatic activation and application of stereotypes (e.g., gender stereotypes) later in life. This processing mechanism is demonstrated by the finding that young people stereotype less than older people (López-Sáez and Lisbona, 2009). This development of stereotyping may be also reflected in the development of the discrepancy between one's own cognitive information processing structure in form of schemata (personal belief) and the perception of attributed characteristics by members of a collective (perceived belief of groups, Macrae et al., 1996). Potentially, this discrepancy results from the fact that young people stereotype less because stereotypes are less developed and because they have more efficient cognitive control processing (Verhaeghen, 2011). This combination of less developed stereotypes and efficient cognitive control might suggest differences, and therefore discrepancies, in the formation as well as the endorsement of a personal belief and the perceived belief of groups (e.g., about the impact of gender on multitasking) particularly in young adults.

### Gender and multitasking

To investigate the different stereotype components, we apply the stereotype of gender effects on multitasking and multitasking performance. In fact, many daily activities involve multitasking when performing two (more or less) simultaneous tasks (e.g., Logan and Gordon, 2001; Salvucci and Taatgen, 2008; Watson and Strayer, 2010; Strobach et al., 2015). This performance is typically effortful and demanding, resulting in a decrement when contrasted with isolated performance of only one task under single-task conditions; as a consequence, using mobile phones when driving is prohibited by law in many countries. This decrement seems to be a universal cognitive phenomenon of human beings (e.g., Pashler, 1994) since its evidence comes from various cultural, societal, and developmental contexts (Strobach et al., 2012a,b) and they are extremely resistant to practice effects (e.g., Van Selst et al., 1999; Strobach et al., 2012c, 2014). Besides these general multitasking characteristics, popular media reports convey an attribution of gender differences to this decrement in multitasking performance (e.g., Shellenbarger, 2003; Pease and Pease, 2003) in that women are better at multitasking than men. Even scientific contributions refer to this popular attribution of gender effects in multitasking (Hambrick et al., 2010; Mäntylä, 2013; Mäntylä and Todorov, 2013; Stoet et al., 2013; Strayer et al., 2013) when, for instance, investigating the amount of time of multitasking spent on household and child care (Offer and Schneider, 2011).

However, to date, there have only been a few empirical studies that have examined gender differences in multitasking (e.g., Hambrick et al., 2010). Therefore, it is still unclear whether the gender stereotype for multitasking is consistent with actual multitasking performance differences. Moreover, the low number of studies on gender effects and multitasking performance have produced inconsistent outcomes in itself: while some studies provided evidence for a gender difference with tendencies of advanced multitasking performance in men (McGowan and Duka, 2000), advanced performance in women (Medland et al., 2002) or mixed results (Saucier et al., 2003; Stoet et al., 2013), others have reported no gender differences at all (Seth-Smith et al., 1989). Recently, Mäntylä (2013) supported the view that women and men are equally good at coping with a multitasking situation including combinations of monitoring and working-memory updating tasks after controlling for spatial abilities (Strayer et al., 2013). Thus, there is no conclusive empirical evidence from well-controlled experimental settings demonstrating that women are superior to men at multitasking.

### The current study

The current study investigates gender stereotyping and multitasking by examining the proportion of gender as a factor that influences multitasking performance reported as a personal belief and as a perceived belief of groups. If the stereotype “gender affects multitasking performance” exists, we firstly hypothesize a substantial number of participants reporting this stereotype^1^. Second, discrepancies between the stereotype's components (i.e., personal belief vs. perceived belief of groups) should be increased in young adults in contrast to older adults. From our view, this assumption is supported by young adults' improved performance to flexibly adapt the cognitive system to changing perspectives and requirements (O'Brien and Hummert, 2006; Verhaeghen, 2011; Wasylyshyn et al., 2011) as being investigated in the sifting paradigm (Kiesel et al., 2010; Strobach et al., 2012d). Third, we hypothesize that the gender stereotype is a specific and independent phenomenon, and thus shows no result pattern that is equivalent for alternative factors potentially influencing multitasking (e.g., difficulty of a multitasking situation, age of a multitasking person, multitasking experience of a person). In this latter case, the gender response pattern is merely the result of participants' general response tendency. Forth, we expect that if people report gender of a person as being a factor that influences multitasking, that they assume superior multitasking performance by women.

## Methods

### Participants

A total of 241 young (*n* = 129) and older (*n* = 112) German individuals participated in this study. The young participants (*M* age = 22.8, *SD* = 2.6, range 19–29) were comprised of 68 women and 61 men and the older participants (*M* age = 70.4, *SD* = 6.3, range 60–85) were comprised of 55 women and 57 men.

### Recruitment procedure

Individuals were recruited through different methods. First, young individuals were recruited through emails on Berlin and Munich university campuses and communities. These emails directed young adults interested in completing a survey on multitasking to a secure online site. Second, older individuals were recruited from senior centers, referrals from students at a large university in Berlin, and an existing university listserv comprised of older individuals interested in participating in research studies. More specifically, the researchers contacted local senior centers in Berlin and recruited older participants to complete a survey and send the completed survey back to the researchers in a prepaid postage envelope. Next, students from a university in Berlin referred participants over the age of 60 to participate in the study in exchange for 2.5% of research credit applied toward their degree requirements. Finally, participants were recruited from an existing university listserv comprised of older adults who are interested in participating in research studies. They were directed to a secure online site where they were able to complete the survey anonymously. All of the older participants were part of a larger study which examined attitudes and behaviors associated with marriage and relationship satisfaction. These data were not relevant to the current study and therefore were not used in the data analyses.

### Multitasking measure

All participants completed two questions pertaining to attitudes toward multitasking after they were provided a short definition of multitasking (i.e., the performance of multiple, simultaneous tasks). First, participants were asked, “What factors do you think in general have an influence on a person's performance in multitasking situations?” (i.e., individual perception). Participants were given the following factors to choose from and were instructed to select all that apply: difficulty of the situation, age of the person, gender of the person, personal experience, and “other”; the order of factor presentation with the exception of “other” was randomized between participants. If a participant selected “gender of the person” as a factor that contributes to a person's multitasking performance, he/she was prompted to then select which gender he/she thinks is better at multitasking situations, women or men. Next, participants were asked, “What factors, in the opinion of the general public (e.g., public, media, etc.), generally have an influence on a person's performance in multitasking situations?” (i.e., public perception); note that we explicitly refer to the “public's” opinion to avoid self-presentational concerns. Again, they were given the same list of factors (i.e., difficulty of the situation, age of the person, gender of the person, personal experience, and “other”) in a randomized order (with the exception of “other”) and asked to check all that apply. If a participant selected “gender of the person,” he/she was prompted to choose which gender he/she felt the general public thinks is better at multitasking situations, women or men. The “other” factor provided rare and unsystematic responses. Therefore, we did not analyze these responses in detail.

## Results

### Existence of gender stereotype

Does the stereotype “gender affects multitasking performance” exist? And if so, is there a substantial number of participants reporting this stereotype. On a descriptive level, 127 (52.7%) participants reported the gender stereotype as a perceived belief of groups whereas 84 (34.9%) participants reported the gender stereotype as a personal belief. These amounts can be considered as being substantial when 11% of reports of “Germans are practical” is a stereotype, too (Katz and Braly, 1933; Krueger, 1996).

### Gender, age, difficulty vs. experience

Are discrepancies between the gender stereotype's components (i.e., personal belief vs. perceived belief of groups) increased in young adults in contrast to older adults? And if so, is this result pattern unique for this stereotype (i.e., is this stereotype specific and independent from the other factors potentially modulating multitasking)? To answer these questions, in our first inferential analyses, we examined whether there was a significant difference between the factors that influence multitasking in the personal belief and perceived belief of groups among young individuals. Because the data were nominal, a series of four McNemar tests were conducted, one for each factor (i.e., gender of the person, age of the person, difficulty of the situation, and personal experience). As indicated in Table 1, gender [*X*^2^_(1, *N* = 129)_ = 18.50, *p* < 0.001] and age of the person [*X*^2^_(1, *N* = 129)_ = 36.16, *p* < 0.001] contributed more to young adults' perceived belief of groups about multitasking, while difficulty of the situation [*X*^2^_(1, *N* = 129)_ = 48.79, *p* < 0.001] and personal experience [*X*^2^_(1, *N* = 129)_ = 55.71, *p* < 0.001] played a larger role in young adults' personal belief of what factors shape multitasking performance.

**Table 1:** **McNemar tests for young and older individuals and factors that contribute to multitasking performance in the personal belief and perceived belief of groups**.

	**Perceived belief of groups**												
		**Gender of person Chi Square**			**Age of person Chi Square**			**Difficulty of situation Chi Square**			**Personal experience Chi Square**		
		**Yes**	**No**	***X*^2^**	**Yes**	**No**	***X*^2^**	**Yes**	**No**	***X*^2^**	**Yes**	**No**	***X*^2^**
**YOUNG INDIVIDUALS**													
Personal	Yes	17	18	18.50^***^	64	5	36.16^***^	58	59	48.79^***^	49	66	55.71^***^
belief	No	56	38		51	9		3	9		3	11	
**OLDER INDIVIDUALS**													
Personal	Yes	41	8	1.57	65	9	2.89	33	14	.16	40	24	1.23
belief	No	15	48		19	19		11	54		16	32	

*** < 0.001.

An equivalent analysis examined whether there was a significant difference between the factors that influence multitasking in the personal belief and perceived belief of groups among older individuals. Again, four McNemar tests were conducted, one for each factor. As reported in Table 1, there were no significant differences among older individuals' reports of which factors contributed to their personal belief about multitasking or what factors they felt influenced the perceived belief of groups of multitasking.

**Figure 1:**
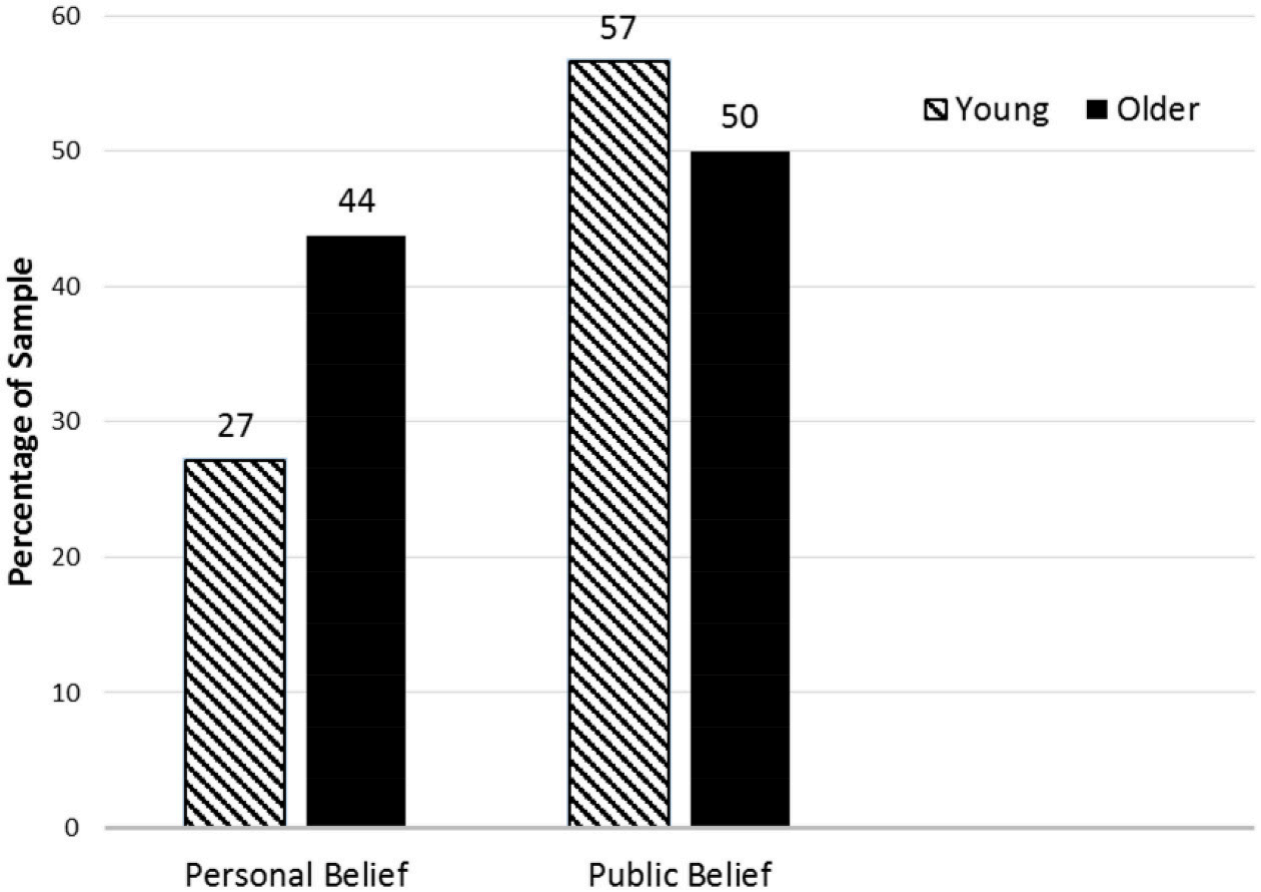
**Gender and multitasking performance**. This figure illustrates the percentages of young and older participants who reported that gender contributes to multitasking performance in personal belief and the perceived belief of groups. Young *n* = 129. Older *n* = 112.

Overall, young individuals reported more discrepancy among the factors that influence multitasking in the personal belief and perceived belief of groups than did older individuals. More specifically, young individuals reported that the age and gender of the person were factors that were more influential to the perceived belief of groups of multitasking whereas difficulty and experience of the situation were factors that they thought contributed more to the personal belief about multitasking. This is particularly illustrated for gender and multitasking in Figure 1. Older individuals did not report that certain factors contributed more or less to personal belief and perceived belief of groups about multitasking situations.

### Men vs. women

**Figure 2:**
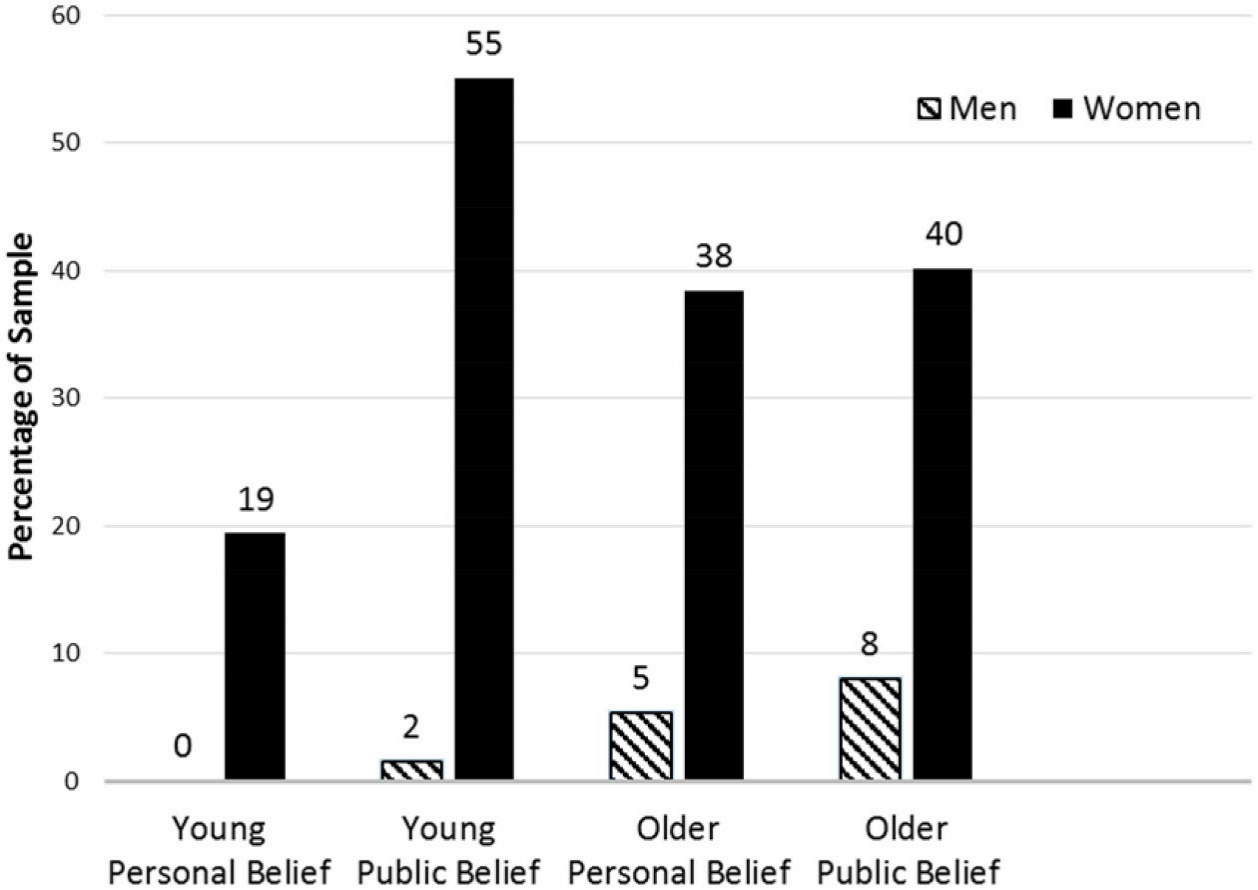
**Group differences in perceptions of gender and multitasking performance**. This figure illustrates percentages of group differences in the discrepancy between personal belief and the perceived belief of groups with regard to whether men or women are better at multitasking. Young *n* = 129. Older *n* = 112.

Finally, we examined which gender was perceived to perform better in multitasking situations. According to a frequency analysis, personal belief for young (100%, *n* = 35) and older (88%, *n* = 43) people as well as and perceived belief of groups for young (97%, *n* = 71) and older (83%, *n* = 45) people favored women as performing better in multitasking situations (Figure 2). Because only a subset of participants answered this question, and thus, small expected values in the 2 × 2 contingency table were anticipated, Fisher's exact tests were used. As indicated in Table 2, the Fisher's exact tests revealed a significant difference between young and older adults' personal belief of which gender performs better in multitasking situations. Specifically, young people (compared to older people) were significantly more likely to report that they personally believed women were more superior at multitasking situations than men (*p* = 0.04, two-tailed) as well as report that, in the perceived belief of groups, women are better multitaskers than men (*p* = 0.01, two-tailed). In sum, women are generally thought to perform better in multitasking situations than men. Further, with regard to the personal belief and the perceived belief of groups, young individuals (compared to older individuals) believe that women perform better in multitasking situations than men.

**Table 2:** **Fisher's exact tests for young vs. older individuals, gender, and personal belief vs. the perceived belief of groups about multitasking performance**.

	**Gender of person**		
	**Men**	**Women**	**Fisher's exact test**
**PERSONAL BELIEF**			
Young	0	35	*p* = 0.04
Older	6	43	
**PERCEIVED BELIEF OF GROUPS**			
Young	2	71	*p* = 0.01
Older	9	45	

## Discussion

This study was designed to examine how gender influences multitasking in people's personal belief and perceived belief of groups. Specifically, we aimed at establishing our intuition of a stereotype that gender has an effect on multitasking performance. Further, we expected that there are discrepancies between two components of stereotyping (i.e., a cognitive information structure vs. the perceptions of attributed characteristics; e.g., Devine, 1989; Stangor and Schaller, 2000; Cohen and Garcia, 2005) and discrepancies in such reports increase with increased cognitive flexibility. As a consequence of their increased flexibility, we assumed that young adults were more likely to show these discrepancies in contrast to older adults. Finally, we predicted that if people reported that gender of a person was a factor that influences multitasking (in personal belief and the perceived belief of groups) they assumed superior multitasking performance in women.

Importantly, we found that a substantial number of participants reported that gender affected multitasking, suggesting that there is in fact a gender stereotype. However, there was a perceived belief of groups of gender effects on multitasking performance in a relatively high number of participants when contrasted with the number of people that reported such effects in one's own perception (i.e., personal belief). The discrepancy between personal belief and the perceived belief of groups is exclusively present in young adults. In tests on the proportion of young adults assuming that one's gender affects multitasking, a relatively low number of individuals reported personal beliefs when contrasted with the report of such perceptions in the perceived belief of groups. In contrast, there was a similar proportion of reports of personal beliefs and the perceived beliefs of groups in older adults. The age difference between young and older adults was reflected in the personal belief, demonstrating a higher number of older adults in contrast to young adults reporting that the gender of a person affected multitasking performance. There was no such age difference with regard to the perceived belief of groups. These findings are consistent with previous literature (e.g., López-Sáez and Lisbona, 2009) that reports reduced discrepancies between the components of stereotyping as one's own cognitive structure and the perception of characteristics attributed by all (or most) individuals of a collective with increased age.

In our hypotheses, we motivated the assumption of increased discrepancies in young adults with their improved ability to shift between different task requirements (i.e., different task sets, e.g., Verhaeghen, 2011). However, there are other alternative explanations which may also account for this effect. For instance, the increased number of reported individual perceptions in older adults may result from generally increased conformation of individual cognitive structures with a stereotype, which develops with age from young to older adulthood. Related, a critical issue of the present study design is an overlap in the factors that participants identified as contributing to their own beliefs about multitasking ability and the perceived belief of groups about multitasking. This overlap could be reflective of either (1) the difficulty moving between one's own perspective and thinking about the perspective held by others (i.e., the collective), or (2) a genuine overlap in one's own perspective on multitasking and the perspective held by others. This overlap may have obscured the present study's data.

Our data also show that the result pattern of the perceived gender effect at multitasking is specific for this gender factor when contrasted with the alternative factors (i.e., difficulty of a multitasking situation, age of a multitasking person, multitasking experience of a person). That is, across analyses, result patterns of the gender factor differed from all other factors. This specificity of gender-factor ratings also excludes the assumption that the result of the gender factor is the result of a general response or reporting strategy.

Nevertheless, women's multitasking was clearly rated as superior in contrast to men's multitasking across the present age samples as well as in the personal belief and the perceived belief of groups. This clear superiority is consistent with the stereotype of gender differences and multitasking. So, previous studies on this issue correctly referred to the attribution of women's superiority when introducing this issue (e.g., Hambrick et al., 2010; Mäntylä, 2013; Stoet et al., 2013; Strayer et al., 2013).

Limitations of the present study may be (1) its lacking explanation of how the perception of stereotypes and individual constructs on multitasking and gender were established, (2) lacking information about factors that may influence stereotyping such as socio-economic status, level of education, and intelligence, and (3) the study's cross-sectional nature. Specifically, future studies could ask about the actual source that promotes the perceived belief of groups on the relationship between multitasking and gender; nevertheless, the preliminary aim of the present study was to establish this relationship before going the next step. Furthermore, the present study approached the perception of gender effects on multitasking from an aging perspective with a cross-sectional design. This design type does not allow us to differentiate between age and time of birth as the factors responsible for age differences. Such a differentiation is rather achievable in a study with a longitudinal design which we leave for future work. Another, promising direction for future studies could be to focus on the personal belief and the perceived belief of groups on gender in the context of alternative cognitive tasks (e.g., spatial cognition; see Ritter, 2004; Halpern et al., 2011).

In sum, the present study demonstrated hints for the existence of the stereotype that gender affects multitasking performance and this influence results in an advantage in women. In fact, the amount of reported attributions of gender effects at multitasking in the perceived belief of groups was relatively high in a survey among young adults, but not in older adults, when contrasted with the amount of reported personal beliefs.

### Conflict of interest statement

The authors declare that the research was conducted in the absence of any commercial or financial relationships that could be construed as a potential conflict of interest.

## Footnotes

^1^Of course, stereotypes are social to the extent that they are shared by members of a group, but the concept implies the assumption of some level of consensus: stereotypes are shared perceptions among group members. However, it is not clear what minimum degree of consensus is required to call a phenomenon as a stereotype (Krueger, 1996).
